# Surgical Treatment of Two Simultaneous De Novo Para-Anastomotic Aneurysms Following Side-to-Side Bypass of Anterior Cerebral Artery: A Technical Case Instruction

**DOI:** 10.1227/neuprac.0000000000000139

**Published:** 2025-05-06

**Authors:** Yulius Hermanto, Gahn Duangprasert, Sergi Cobos Codina, Kosumo Noda, Nakao Ota, Rokuya Tanikawa

**Affiliations:** * Department of Neurosurgery, Stroke Center, Sapporo Teishinkai Hospital, Sapporo, Japan;; ‡ Department of Neurosurgery, Dr. Hasan Sadikin Hospital, Bandung, West Java, Indonesia

**Keywords:** De novo aneurysm, Para-anastomotic aneurysm, In situ bypass, Modified trapping, Anterior cerebral artery aneurysm

## Abstract

**BACKGROUND AND IMPORTANCE:**

Anterior cerebral artery aneurysms requiring bypass are rare; thus, the literature regarding the subsequent pathology related to the procedure is severely lacking. Hence, there is no consensus regarding the management strategy. The progressive enlargement of de novo para-anastomotic aneurysm carries a higher rupture risk of this abnormal vessel. The peculiar location, related vascular territory, and small corridor of interhemispheric fissure pose challenges to its management.

**CLINICAL PRESENTATION:**

We present a 70-year-old woman with two simultaneous de novo para-anastomotic aneurysms after an A3-A3 bypass. Previously, she had an A3-A3 bypass and aneurysm trapping of left A2 dissecting aneurysm 7 years ago. On routine follow-up imaging, she had progressive enlargement of two aneurysm-liked lesions on top of anastomosis vessels. She underwent a modified trapping and superficial temporal artery-anterior cerebral artery bypass with excellent clinical outcome and no recurrence.

**CONCLUSION:**

Managing de novo aneurysm formation after in situ A3-A3 bypass is challenging. Complex procedures are required to secure the aneurysm from circulation and ensure its vascularization at the distal site. Long-term follow-up is necessary for all bypass procedures.

**ABBREVIATIONS:**
AAA: abdominal aortic aneurysm
DNPA: de novo para-anastomotic aneurysm
ICG: indocyanine green
STA: superficial temporal artery.

Anterior cerebral artery (ACA) bypass is often reserved for managing complex aneurysms because conventional clipping, endovascular coiling, and flow diversion are effective for aneurysm occlusion.^1-4^ This technique aims to restore flow to territories distal to the aneurysm and occlude the aneurysm without ischemic complication.^1-6^ Despite numerous reports^1-8^ regarding the versatility of ACA bypass for aneurysms, there is a lacking literature on the management of de novo pathology after in situ ACA bypass.

De novo para-anastomotic aneurysm (DNPA) formation occurs at varying incidences (4%-11%) after aneurysm treatment.^9,10^ The mechanism of DNPA is not completely understood. Changes in cerebral hemodynamics after arterial occlusion have been associated with the increased incidence of DNPA.^9-11^ In this study, we first reported simultaneous DNPA formation at two sites of side-to-side anastomosis vessels after 4 years of treatment that enlarged on follow-up and underwent surgical treatment with modified trapping and vascular reconstruction.

## CLINICAL PRESENTATION

A 70-year-old woman was admitted to our hospital because of the enlargement of an aneurysm-liked lesion on top of anastomosis vessels during a routine follow-up of imaging; there were no symptoms. She had a history of rupture dissecting aneurysm of left A2, with World Federation of Neurosurgical Societies grade 3 around 7 years ago, the computed tomography scan revealed a Fisher grade 2 subarachnoid hemorrhage (**Supplemental Digital Content 1**, http://links.lww.com/NS9/A41) and subsequent computed tomography angiography (CTA) found a 6-mm dissecting aneurysm at left A2 (**Supplemental Digital Content 1**, http://links.lww.com/NS9/A41). In her first admission at another hospital, she had surgery for an aneurysm clipping; however, owing to an unsuccessful A3-A3 bypass, the aneurysm was not secured.

**FIGURE 1.**
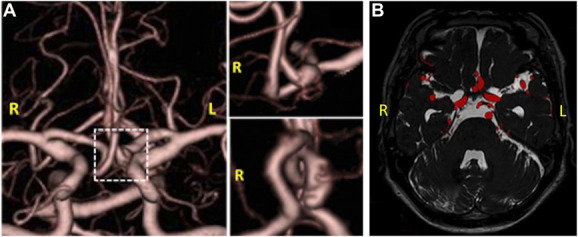
**A**, Follow-up computed tomography angiography revealed the left A2 aneurysm and presumptive failed A3-A3 bypass. **B**, Fusion MRI image confirmed the aneurysm and no sign of thrombosis.

Three months later, she was referred to our hospital with negligible neurological deficits. The follow-up CTA revealed the left A2 aneurysm and presumptive failed A3-A3 bypass (Figure 1A), and the fusion MRI image confirmed the aneurysm and did not find any sign of thrombosis (Figure 1B). We conducted aneurysm trapping with A3-A3 side-to-side bypass. Intraoperative indocyanine green (ICG) and postoperative CTA showed the patency of bypass as well as successful trapping of the aneurysm (Video 1). She was discharged uneventfully and routinely controlled.

**FIGURE 2.**
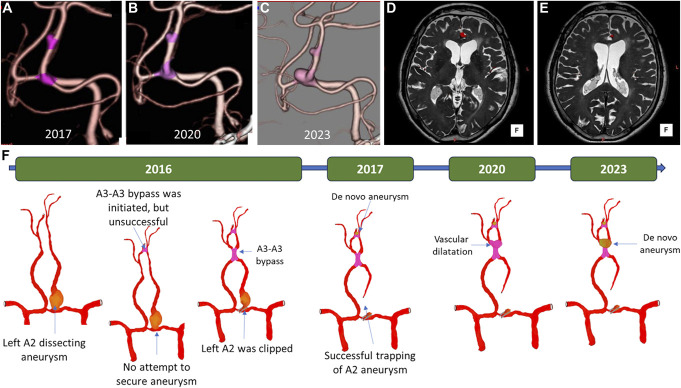
**A**, Aneurysm-like lesion at the failed attempted A3-A3 bypass. **B**, Dilatation at the site of our A3-A3 bypass. **C**, De novo aneurysm formation at the distal site of our A3-A3 bypass. **D** and **E**, There was no thrombosis at both aneurysm sites. **F**, Schematic illustration of clinical progression of the patient's vascular pathology.

On routine follow-up CTA, we observed an aneurysm-like lesion at the failed attempted A3-A3 bypass on 1 year after the surgery (Figure 2A). In the subsequent CTA, there was a dilatation at the site of our A3-A3 bypass 3 years later (Figure 2B), and we closely monitored the patient. In addition, 3 years later, she remained asymptomatic but there was DNPA at the distal site of our A3-A3 side-to-side bypass (Figure 2C). The MRI fusion images showed no thrombosis at either aneurysm site (Figure 2D and 2E). A schematic illustration of clinical progression is presented in Figure 2F; after discussion, we decided to operate on the patient.

**FIGURE 3.**
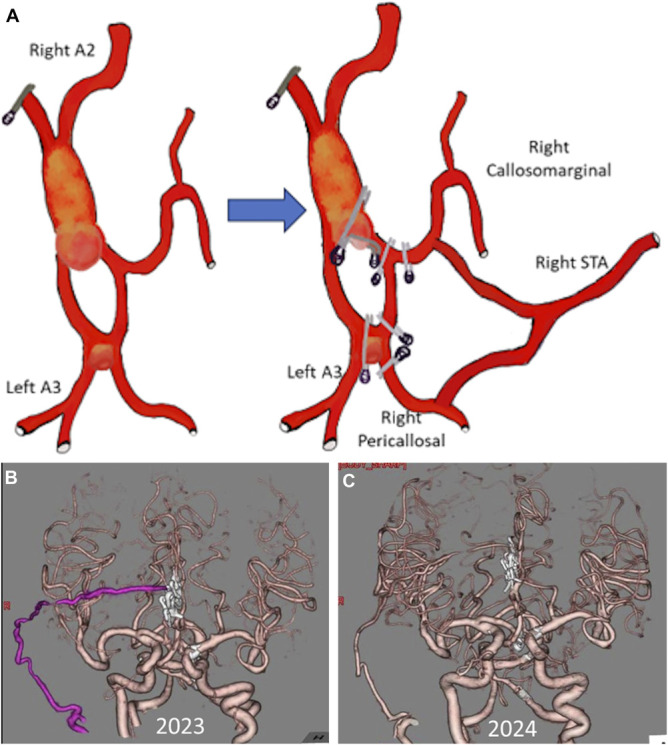
**A**, Schematic illustration of modified trapping and reconstruction of right distal anterior cerebral artery with Y-shaped superficial temporal artery bypass. **B**, Immediate postoperative CTA. **C**, 1-year postoperative CTA. CTA, computed tomography angiography.

We performed modified trapping and bypass for distal ACA circulation with the right superficial temporal artery (STA) as the donor artery. The surgery was conducted through a bifrontal interhemispheric approach; to achieve adequate exposure, the retraction of both cingulate gyri was necessary. After meticulous dissection, a DNPA was observed at the distal of our previous A3-A3 bypass; initially, we attempted to secure the aneurysm by clipping at the aneurysm neck. Further dissection revealed another aneurysm at the first A3-A3 bypass. STA-ACA bypass was performed to vascularize both distal ACAs and isolate the aneurysm from ACA circulation. The flow to the distal left ACA was determined to be sufficient; hence, the one STA donor branch was moved from left A3 to the right callosomarginal artery. Then, modified trapping was performed, and both aneurysms were isolated from the circulation. The intraoperative ICG confirmed the flow to both distal ACAs, and the DWI showed no ischemic complication (Video 2). The reconstructed flow for the right pericallosal and callosomarginal arteries was from STA donor, while the left ACA was from the right ACA (Figure 3A).

The patient developed a mild cognitive deficit postoperatively that recovered over time, possibly because of retraction of both cingulate gyri during the surgery. After 1-year follow-up, the patency of the graft was confirmed with no recurrence of aneurysm formation (Figure 3B and 3C), and she also had no neurological deficit.

The patient consented to the procedure and the publication of her images. Institutional review board approval was not required.

Discussion on microvascular anastomosis remains a relevant armamentarium for neurosurgeons in the management of complex ACA aneurysms, particularly those located on distal arterial segments where collateral flow is less defined.^2-7,11^ Vascular reconstruction with intracranial arteries that are in the surgical field is favorably rather than classical external carotid-internal carotid bypass; hence, A3-A3 in situ bypass is a common option for distal revascularization.^5-7,11-13^

The occurrence of the DNPA has been reported after coronary bypass,^14,15^ open surgery of abdominal aortic aneurysm (AAA),^16,17^ femoropopliteal bypass,^18^ and high flow internal carotid bypass.^19^ Several groups also noted the occurrence of DNPA after classical STA-MCA bypass.^20-26^ The true incidence of intracranial DNPAs is perhaps underreported; according to the data from AAA bypass surgery, the expected incidence ranges from 2% to 3.6% during a 10-year follow-up.^16^ The mechanism behind de novo DNPA is poorly understood. The histopathological study showed a neointima proliferation at the anastomotic site, suggesting a vascular remodeling due to shear stress.^20,27^ The previous study demonstrated a high wall shear stress that coincident with the pulsation in the front wall of side-to-side ACA anastomosis where an aneurysm developed.^28^ In our case, two DNPAs occurred simultaneously after side-to-side A3-A3 bypass; there was no artificial bifurcation created by the procedures; perhaps the disruption of the intimal layer or internal elastic layer during anastomosis because of the intraluminal suturing phase of side-to-side bypass and exposure to high wall shear stress might contribute to aneurysm formation.^13,25,28^ The identified modifiable risk factors for DNPAs from the AAA study are age, hypertension, ischemic heart disease, and cerebral vascular disease.^16^ Suggesting chronic endothelial stress might contribute to DNPA formation.^9^

The nature course of intracranial DNPA is not well studied; however, two studies reported hemorrhage from the DNPA,^29,30^ suggesting that treatment is needed, particularly for enlarging DNPA. All previous studies suggest open surgery for the treatment of DNPAs, either clipping or aneurysmectomy.^19,21-26,28^ In this study, we opted for modified trapping and STA-ACA bypass to revascularize the right distal A3 territory and reduce hemodynamic stress on side-to-side bypass.

### Key Results

This study illustrates the rare occurrence of two simultaneous DNPAs after in situ A3-A3 bypass 7 years postanastomosis. The patient's presentation of progressive enlargement of DNPAs carries a higher rupture risk of this abnormal vessel. The DNPA location and small corridor of interhemispheric fissure pose challenges to its management. This condition only suitable for the interhemispheric approach, which requires retraction of both cingulate gyri and protection of olfactory nerves, but it offers unparalleled exposure of the anatomy and possibility of distal revascularization.

### Limitations

This report has several limitations. It was a single case. Despite a successful intervention outcome, additional cases are essential to identify the risk factors and mechanisms behind the occurrence of intracranial DNPAs. The revascularization strategy may depend on the aneurysm location and the surgeon's preference, yet the goal is assurance of distal vascularization.

## CONCLUSION

Managing de novo aneurysm formation after in situ A3-A3 bypass is challenging. Complex procedures are required to secure the aneurysm from circulation and ensure its vascularization at the distal site. Long-term follow-up is necessary for all bypass procedures. This study will spark further investigation to identify risk factors and prevent this troublesome pathology, as microvascular anastomosis remains evolving and is needed to treat complex aneurysms in the growing endovascular era.

## SUPPLEMENTAL DIGITAL CONTENT

**Supplementary Figure 1.** A) Initial presentation of the patient with subarachnoid hemorrhage Fisher grade 2. B) CTA showed a dissecting aneurysm of left A2 (pointed by white arrow).

## COMMENTS

This case report describes a rare and challenging neurovascular pathology—the development of two simultaneous de novo para-anastomotic aneurysms (DNPAs) following A3-A3 in situ bypass. The authors' documentation of this phenomenon and their surgical management adds valuable knowledge to the limited literature on postbypass complications in the anterior cerebral artery territory. The progressive enlargement of DNPAs following cerebrovascular bypass represents a significant therapeutic challenge, particularly in the narrow confines of the interhemispheric fissure. The authors' approach—modified trapping and STA-ACA bypass—demonstrates an elegant solution for securing the aneurysms while maintaining adequate distal flow. Their technique balances the dual imperatives of excluding the aneurysms from circulation while preserving perfusion to critical territories. The authors' thorough documentation of the aneurysms' evolution over 7 years is of particular value, providing insight into the natural history of this rare complication. The schematic illustrations demonstrate the hemodynamic changes and surgical strategies employed, enhancing the educational value of this technical case instruction. The report appropriately acknowledges the neurological consequences of surgical manipulation within the interhemispheric fissure, noting the patient's transient cognitive deficits likely related to cingulate gyri retraction. This honest assessment of surgical morbidity, alongside the successful aneurysm treatment, offers a balanced perspective on the risk-benefit considerations in complex cerebrovascular surgery.

As microvascular bypass techniques remain essential in managing complex cerebrovascular pathologies despite advances in endovascular therapy, this report serves as a necessary reminder of the need for long-term surveillance following these procedures. The case underscores that vascular remodeling at anastomotic sites can occur years after apparently successful treatment.

**Clemens M. Schirmer**

*Wilkes-Barre, Pennsylvania, USA*

## Notes

First of all, the medical team should be commended for their nice, if not heroic, surgical performance.

Such surgery, deep inside the interhemispheric fissure, surely exposes to postoperative cognitive deficits that may impair the patient's practical daily-life. The way to work for a long duration under retraction of both anterior cingula, has been considered in literature as for example in a Alvernia et al's article (Is exposure of the superior sagittal sinus necessary in the interhemispheric approach? Neurosurgery, 2009, 65 (5): 962-964).

At a time when the endovascular methods for treating aneurysms have become predominant, such a report brings attention on the necessity to maintain open microsurgical techniques available in well-trained hands.

**Marc Sindou**

*Lyon, France*
